# Long-Term Follow-Up for Child with Ataxia Telangiectasia Related Scoliosis Using Surface Topography: A Case Report

**DOI:** 10.3390/reports9010093

**Published:** 2026-03-23

**Authors:** Brian Wagner, Adam Thiessen, Xue-Cheng Liu

**Affiliations:** 1 School of Medicine, Medical College of Wisconsin, Milwaukee, WI 53226, USA; 2 Department of Orthopaedic Surgery, Children’s Wisconsin, Milwaukee, WI 53226, USA

**Keywords:** ataxia telangiectasia, scoliosis, surface topography, case report

## Abstract

**Background and Clinical Significance:** Scoliosis prevalence in patients with ataxia telangiectasia (AT) is higher than in the general population. Scoliosis monitoring is traditionally performed using X-rays, but radiographic imaging is contraindicated in AT patients due to radiation sensitivity. Current guidelines suggest a diagnostic radiograph with subsequent Magnetic Resonance Imaging (MRI). In this report, we (1) evaluated the feasibility of using surface topography (ST) to observe 3D spine curvature trends in a long-term follow-up of a patient with scoliosis and AT, and (2) developed a novel paradigm for monitoring scoliosis in AT patients. **Case presentation:** A female patient (11 years old) with AT and scoliosis was monitored using ST in five visits over four years. Between subsequent visits, her ST measurements included average changes in thoracic scoliotic angle of 5.5° ± 4.9°, thoracolumbar scoliotic angle of 7.8° ± 5.5°, thoracic axial surface rotation (ASR) of 8.0° ± 8.5°, thoracolumbar ASR of 7.0° ± 4.5°, thoracic apical deviation of 6 mm (only measured in two visits), thoracolumbar apical deviation of 10 mm ± 2.4 mm, pelvic obliquity of 5.8 mm ± 3.9 mm, shoulder obliquity of 20 mm (only measured in two visits), coronal imbalance of 11.8 mm ± 9.7 mm, and kyphotic angle of 5.5° ± 5.4°. ST effectively monitored curve patterns throughout the 4-year treatment period, enabling informed treatment decisions by the provider, patient, and family. We also developed a novel paradigm combining diagnostic MRI with serial ST imaging every 6–12 months to monitor curve progression with supplemental MRI as needed. **Conclusions:** Our novel ST paradigm provides a feasible method for monitoring 3D scoliosis progression in AT patients while avoiding unnecessary radiographic imaging.

## 1. Introduction and Clinical Significance

### 1.1. Ataxia Telangiectasia and Radiation

Ataxia telangiectasia (AT), also known as Louis-Bar Syndrome, is a rare, autosomal recessive disorder caused by a mutation of the Ataxia Telangiectasia Mutated (ATM) gene on chromosome 11. AT may affect as many as one in 90,000 births to as few as one in 333,000 births in the US [1]. ATM is a nuclear protein kinase that is in the complex signaling pathway involved in double-stranded DNA repair [2]. Therefore, the genetic integrity of cells in patients with AT is highly susceptible to radiation [3,4]. Radiation susceptibility can manifest as further telangiectasia, fibrosis, and cellular toxicity [5]. Unfortunately, high doses of radiation in patients with AT may rapidly culminate in dire clinical outcomes, including death [3,6]. Patients with AT are 100 times more likely to be diagnosed with cancer than those without AT [7].

### 1.2. Scoliosis in Ataxia Telangiectasia

While scoliosis is not as strongly associated with AT compared to other ataxic disorders such as Friedreich’s Ataxia [4], scoliosis may be seen in up to 17% of patients with AT [3] and is most associated with late-onset AT [8]. Furthermore, family members of patients with AT are 2 to 5 times more likely than the general population to be treated for severe scoliosis [9].

While therapeutic radiation is understood to pose a significant risk to patients with AT, there is little documentation regarding diagnostic radiation. As such, current guidelines suggest that a one-time diagnostic radiograph be used for the diagnosis of scoliosis, with follow-up scans performed via MRI rather than X-ray in order to avoid ionizing radiation [3].

### 1.3. Surface Topography System

Surface topography (ST) uses light reflection to obtain a topographic analysis of scoliosis patients’ backs to calculate a variety of physical characteristics, including scoliotic angle, which is an estimate of the coronal Cobb angle. The ST also estimates axial surface rotation (ASR), which is an estimate of the axial vertebral rotation (AVR) as determined by 3D radiographic reconstruction. While not equivalent, the topographic scoliotic angle is highly correlated with the Cobb angle [10,11], and ASR is moderately correlated with AVR [12]. As such, topographic analysis of scoliosis is a reliable method in the surveillance of scoliosis patients while minimizing radiation exposure via repeat radiographs.

To our knowledge, there are no published studies that report the effective monitoring of scoliosis in AT patients without initial radiography and subsequent MRIs. The goals of this case report are: (1) to evaluate the feasibility of using ST to measure 3D spine curvatures in a long-term follow-up of a patient with scoliosis and AT, and (2) to develop a diagnostic paradigm in the use of surface topography for AT-related scoliosis.

## 2. Case Presentation

### 2.1. Materials and Methods

A patient diagnosed with AT and scoliosis was seen in five clinic visits over the course of four years. At each of these visits, clinical physical examination and surface topography data were collected. Given the patient’s ataxia, bilateral stabilizing handles were required during ST analysis to prevent postural sway. One to three measurements were taken at each visit; the number of measurements from which we collected data was chosen based on the patient’s standing tolerance and steady position. When multiple scans were obtained at a single visit, the scan with the most upright and stable posture was selected. If all three scans were selected, then the median value was considered. Two members of the research team individually identified which scans would be used, and any discrepancies between selections were resolved via discussion.

Surface topography was measured using the DIERS 4D Formetric surface topography system (DIERS Biomedical Solutions, Wiesbaden, Germany). All measurements were obtained by the same individual to ensure consistency in methodology and technique. While we did not measure our intra-rater reliability, prior literature suggests excellent intra-class correlation coefficients with a strong correlation between Cobb angle and scoliotic angle [11,13]. A total of seven variables were reported. Scoliotic angle was obtained as a proxy for radiographic Cobb angle. Apical deviation was measured as the horizontal distance between the expected vertebral midline and the actual position of the apical vertebrae of the major curve. Pelvic obliquity was measured in all five visits, while shoulder obliquity was only measured in visits four and five. Coronal imbalance, the horizontal distance between a vertical C7 plumbline and a central sacral vertical line, was also measured. Axial vertebral rotation was measured along the vertical length of the spine. The maximum kyphotic angle was measured as the angle between the surface tangents of the inflection point in the cervical-thoracic region and the inflection point of the thoracolumbar region. Depending on the measurement in question, the DIERS 4D Formetric system has a within-day smallest detectable change (SDC) ranging from 1.8 to 25.03 units for a single scan and from 0.97 to 17.93 units for 6 scans, while between-day SDC ranged from 1.44 to 28.24 and 1.05 to 22.2 units for a single and 6 scans, respectively [13]. The standard error of measurement (SEM) for ST is considered to be of high measurement stability for 34 of 40 variables for same-day measurements and for 31 of 40 variables between days [13], while the intra-rater SEM for scoliotic angle over the span of 1 week was 5.2° [11].

### 2.2. Clinical Case Overview

Dawn (pseudonym) was diagnosed with AT at 3 years old. At age 11 years, Dawn’s primary care provider (PCP) noted spinal curvature on a routine physical exam. Her PCP later noted curve progression and subsequently referred Dawn to the Orthopedics clinic for evaluation at age 13 years. At Dawn’s initial evaluation in the Orthopedics clinic, the seated forward bending test showed left-sided thoracolumbar prominence. Dawn was unable to perform a forward bend during her second and third visits due to ataxia. At her fourth and fifth visits, she was able to perform the maneuver, which demonstrated right thoracic and left lumbar prominences. Upon sitting evaluation in the clinic, her initial visit showed left shoulder elevation, absent pelvic obliquity, a right flank crease, and normal thoracic kyphosis and lumbar lordosis. No abnormalities were seen at her second visit, though her third visit indicated a return of the mild right flank crease. Due to her AT diagnosis, diagnostic radiographs were not obtained. Instead, ST imaging was used to monitor her scoliosis.

**Figure 1:**
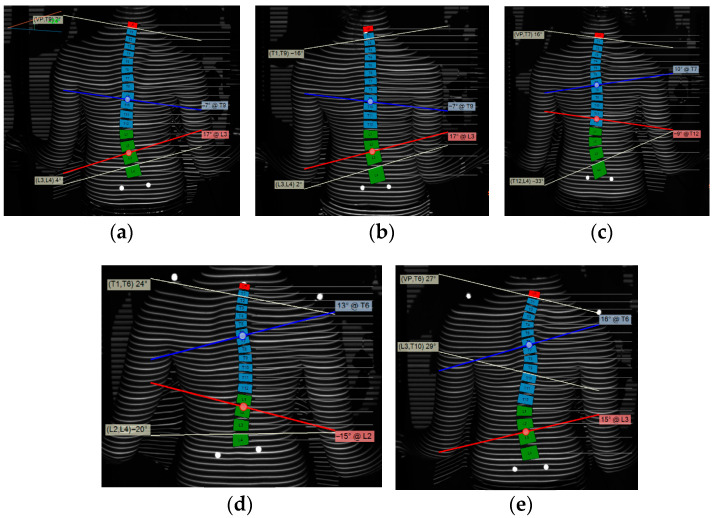
Coronal plane topographic analysis via DIERS 4D Formetric system. The curves are traced in blue and red lines for panels (**a**,**b**), the blue and red lines for the thoracolumbar curve and the red and white curve for the lumbar curve in panels (**c**,**d**), and the blue and white lines forming the thoracic curve and the white and red line forming the thoracolumbar curve in panel (**e**). (**a**) Visit one in September 2020. ST shows 24° of levoscoliosis. (**b**) Visit two in April 2021. ST shows 24° of levoscoliosis. (**c**) Visit three in January 2022. ST shows 33° of levoscoliosis. A dextroscoliosis in the thoracic region (19°) was initially developed. (**d**) Visit four in December 2022. ST shows 28° of dextroscoliosis. A second curve shows 20° of levoscoliosis. (**e**) Visit five in June 2024. ST shows 30° of dextroscoliosis. A second curve shows 29° of levoscoliosis.

Nighttime Providence bracing was prescribed at her first orthopedic visit to prevent curve progression, and Dawn’s mother later reported approximately 10 h of bracing per night with no complaints. Brace compliance was not verified using sensors or other objective means. Given relative curve stability on ST at her fourth visit (Figure 1) and presumptive skeletal maturity based on age (15 years) and being 3 years post-menarche, it was recommended that she wean from her brace in 6 months and receive follow-up ST analysis in 3–6 months after weaning. Importantly, skeletal maturity was not confirmed via objective means such as Risser’s sign or Sander’s stage, given the contraindication to radiographic imaging in this patient.

Dawn wore ankle-foot orthotics and transferred independently to and from her wheelchair and had been engaged in weekly physical therapy since age 11 years. The primary focus of this treatment was to improve general strengthening, mobility, and balance in her daily life; Dawn did not undergo physiotherapeutic scoliosis-specific exercise treatment (PSSE).

### 2.3. Surface Topography

As measured by ST, Dawn’s major curve was best described as levoscoliosis of the thoracolumbar region with a scoliotic angle between 24° and 30° over the first three visits. The major curve shifted to have a rightward apex primarily in the thoracic region with a scoliotic angle between 28° and 30° for her final two visits (Figure 1). The apical deviation varied at each visit, though the thoracolumbar apical deviation (i.e., the region of the major curve in Dawn’s first three visits) was greater than the thoracic apical deviation (i.e., the region of the major curve in her last two visits) (Table 1). Pelvic obliquity decreased from 6 mm to 1 mm over the first three visits before increasing to 11 mm at her fourth visit (Table 1). Shoulder obliquity increased between her third and fourth visits, but was unable to be measured at the first three visits (Table 1). Finally, coronal imbalance varied at each visit, starting at −3 mm and becoming progressively more positive through the last visit (+14 mm) in all but the second visit (−20 mm) (Table 1).

Clinical exam indicated loss of thoracic kyphosis at Dawn’s fourth and fifth visits. This conclusion was supported by ST, with a maximum kyphotic angle of 9° and −4°, respectively, down from 12° at her first visit (Table 1).

**Figure 2:**
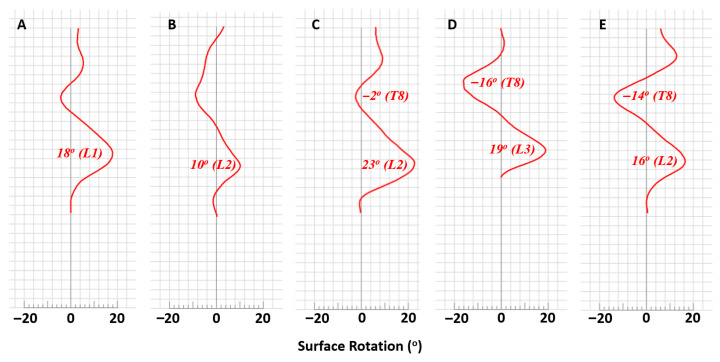
Surface topography estimations of ASR throughout the vertical course of the spine. Landmark vertebrae are indicated on the y-axis. Positive values indicate rotation in the counterclockwise direction from a superior to inferior perspective. Maximum ASR is reported at the region of the major curve. (**A**) Visit one in September 2020. Maximum ASR of 18° at L1. (**B**) Visit two in April 2021. Maximum ASR of 10° at L2. (**C**) Visit three in January 2022. Maximum ASR of −2° at T8 and 23° at L2. (**D**) Visit four in December 2022. Maximum ASR of −16° at T8 and 19° at L3. (**E**) Visit five in June 2024. Maximum ASR of −14° at T8 and 16° at L2.

In the transverse plane, maximum ASR (reported in the region of the major curve) is indicated as a positive angle if the vertebrae rotate counterclockwise from a superior to inferior perspective. In her first three visits, Dawn’s max ASR varied, starting at 18°, shifting to 10°, then increasing to 23°, all in the L1–L2 region of her thoracolumbar major curve (Figure 2).

## 3. Discussion

Throughout five visits over four years, 3D trunk data were successfully registered and analyzed for reliable comparisons, with subsequent visits showing average changes in the thoracolumbar scoliotic angle of 7.8° ± 5.5° from visit one through visit five, thoracic scoliotic angle of 5.5° ± 4.9° from visit three through visit five, apical deviation of 10 mm ± 2.4 mm in the thoracolumbar curve from visits one through five and 6 mm in the thoracic curve between visits four to five (the apical deviation of the thoracic curve was not measured at visit three), magnitude of ASR of 7.0° ± 4.5° in the thoracolumbar curve from visits one through five and of 8.0° ± 8.5° in the thoracic curve from visits three through five, pelvic obliquity of 5.8 mm ± 3.9 mm, shoulder obliquity of 20 mm (measured in the fourth and fifth visits only), coronal imbalance of 11.8 mm ± 9.7 mm, and kyphotic angle of 5.5° ± 5.4°. The ST findings support that Dawn’s thoracolumbar major curve transitioned to a double major curve between the second and fourth clinic visits. This change in curve region and direction is plausibly explained by overcorrection due to Providence bracing or postural changes, which are characteristic of the nature of her scoliosis (Figure 1). An alternative explanation is variability in ST imaging, though this is less likely given the aforementioned published reliability of ST and the consistency in methodology and technique afforded by having the same provider perform the scan at each visit. Overall, Dawn’s case was successfully monitored using ST technology.

Our first goal was to evaluate the feasibility of using surface topography in the long-term management of a patient with both scoliosis and AT as a comparison to initial radiograph imaging with subsequent MRI scans [3]. Ataxia and other forms of postural stability can limit the feasibility of surface topography. Dawn was positioned with both hands on the rails. This provided the support and stability necessary for surface topography analysis. It is already known that ST provides accurate scoliosis-related measurements. That is, while not a perfect recreation of radiographic imaging, ST–measured scoliotic angle correlates with radiographic Cobb angle [10,11] and ST-measured ASR correlates with radiographic AVR [14]. ST also provides consistent measurements between same-day and different-day measurements [11,13]. In conjunction with our findings of the feasibility of using ST in patients with AT, these results support the applicability of ST methodology for diagnosing and monitoring scoliosis in patients with radiation sensitivity.

Analyzing the curve in three dimensions permits monitoring of the scoliotic angle as well as other parameters in the coronal, sagittal, and transverse planes. Specifically, reliable and consistent findings in ST data help monitor and identify changing curve patterns throughout the treatment period and allow the provider, patient, and family to make informed treatment decisions. Finally, ST provided adequate acquisition of clinically useful data with no complaints from the patient or her caretakers.

Furthermore, our paradigm provides high cost and time savings to patients and the healthcare system. MRI scans for scoliosis in 2021 cost $2000 to over $22,000, with an average cost of nearly $18,000 [14]. This represents a high cost to the healthcare system. In comparison, the average cost for scoliosis-specific radiographs is between $100 and $140 [15]. Adjusted for inflation from 2000 to 2021 to better compare to the reported cost of MRI scans, radiographs range from $160 to $225 [16], or roughly 1% of the cost of an MRI. In our hospital system, MRI and radiographic imaging are priced similarly to the costs cited above. Conversely, ST scans at our institution were previously charged at a rate equal to single plane radiographic imaging if the patient had to pay out of pocket, but are now included in the clinical visit fees. Additionally, full-spine MRIs have a duration of roughly 1 h and require an hour and a half of anesthesia [17] while EOS radiographic imaging for scoliosis requires 5 min on average [18]. With our team of experienced clinicians and providers, a full ST scan takes 6 s plus approximately 5 min for patient preparation and positioning.

**Figure 3:**
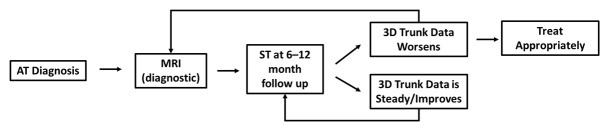
Scoliosis in patients with AT requires radiation-free diagnosis and monitoring. We suggest the initial diagnosis to be made with an MRI, with subsequent imaging using surface topography every 6–12 months. If ST 3D trunk data indicate curve progression, repeat imaging with MRI to confirm progression may be warranted. If ST 3D trunk data indicates curve stability/improvement, no further workup is needed beyond subsequent monitoring using ST.

Our second goal was to synthesize a diagnostic paradigm (Figure 3) to guide future treatment of scoliosis patients with radiation sensitivity as a possible alternative to the current algorithm of one diagnostic radiograph followed by serial MRIs to monitor progression [3]. We suggest that patients receive an initial diagnostic MRI scan followed by surface topography scans every 6–12 months to monitor for curve progression. If ST 3D trunk data suggests a worsening curve compared to previous scans, repeat MRI scans may be used for confirmation. This ST-driven regimen provides data analogous to that obtained from radiographs in patients with idiopathic scoliosis [10,11,12] without exposing the patient to ionizing radiation [3,4,5,6].

Given the ataxic nature of AT, we suggest that patients reduce postural swaying by holding onto handrails or remaining seated during ST scans. When reporting data for clinician review, it is important to include 3D data, including scoliotic angle, maximum apical deviation, coronal imbalance, pelvic and shoulder obliquity, kyphosis, lordosis, and max ASR. The physician can then use available data to formulate a treatment plan involving bracing, PSSE, referral to physical medicine and rehabilitation for personalized wheelchair fitting, or MRI scans if needed to confirm curve progression.

## 4. Conclusions

Over the course of four years, surface topography was used to follow scoliosis in a patient with AT without exposing her to ionizing radiation and without the time and financial cost of serial MRIs. The ST method proffers a practical method to serve as the basis of a diagnostic and management paradigm for patients with concomitant AT and scoliosis, with 3D variables informing treatment and future monitoring of patients’ curves. Treatment for patients may include the implementation of a nighttime brace, personalized wheelchairs with custom padding for posture control, or PSSE. Surface topography may also help guide brace weaning at the point of curve stability or at the point of expected skeletal maturity. Although one-time radiographs followed by MRI are a plausible option to monitor scoliosis in AT patients [3], we conclude that ST offers a feasible, radiation-free, low-cost, and rapid alternative to radiographic spine images to monitor scoliosis in patients with AT-related scoliosis.

## 5. Strengths and Limitations

The strengths of this study include its novelty, as it is the first documented instance of using ST to monitor scoliosis progression in a child diagnosed with AT for avoiding radiation exposure. Additionally, this methodology synthesizes a diagnostic paradigm to guide management of scoliosis patients with radiation sensitivity. However, the study is limited by its single-patient design, with the inclusion of just one patient with AT and scoliosis, and the lack of direct comparison to MRI. Furthermore, while ST is well-studied in AIS, we are unaware of any studies that investigate its use in neuromuscular scoliosis populations.

## Figures and Tables

**Table 1:** ST-based 3D trunk data for 5 visits. Grey background indicates column title.

Visit	Curve Direction	Levels	Scoliotic Angle (°)	Apex Level	Apical Deviation (mm)	Pelvic Obliquity (mm)	Shoulder Obliquity (mm)	Coronal Imbalance (mm)	Maximum Kyphotic Angle (°)
1	Levoscoliosis	T9–L3	24	L1	27	6	not measured	−3	12
2	Levoscoliosis	T9–L3	24	L2	15	3	not measured	−20	13
3	Dextroscoliosis Levoscoliosis	T7–T12 T12–L4	19 33	L2	22	1	not measured	5	7
4	Levoscoliosis Dextroscoliosis	L2–L4 T6–L2	20 28	T8	8	11	22	5	9
5	Levoscoliosis Dextroscoliosis	T10–L3 T6–T10	29 30	T8	2	3	42	14	−4

## Data Availability

Our data supporting the reported results are available and can be provided with archived datasets from our institution upon request.

## Abbreviations

The following abbreviations are used in this manuscript:

ASR	Axial surface rotation
AT	Ataxia telangiectasia
ATM	Ataxia telangiectasia mutated gene
AVR	Axial vertebral rotation
MRI	Magnetic resonance imaging
PCP	Primary care provider
PSSE	Physiotherapeutic scoliosis-specific exercises
ST	Surface topography
